# Metabolomic Markers of Storage Temperature and Time in Pasteurized Milk

**DOI:** 10.3390/metabo11070419

**Published:** 2021-06-25

**Authors:** Kara M. Edwards, Aishwarya Badiger, Dennis R. Heldman, Matthias S. Klein

**Affiliations:** Department of Food Science and Technology, The Ohio State University, Columbus, OH 43210, USA; karamedwards@gmail.com (K.M.E.); badiger.1@buckeyemail.osu.edu (A.B.); heldman.20@osu.edu (D.R.H.)

**Keywords:** NMR, metabolomics, milk, shelf-life, food waste

## Abstract

The current date labeling system for pasteurized milk is based on the predicted growth of spoilage microorganisms, but inherent inaccuracies and the inability to account for environmental factors (e.g., temperature fluctuations) contribute to household and retail food waste. Improved shelf-life estimation can be achieved by monitoring milk quality in real-time. In this study, we identify and quantify metabolites changing over storage temperature and time, the main factors affecting milk stability. Pasteurized 2% fat milk was stored at 4, 10, 15, and 20 °C. Metabolite change was analyzed using untargeted and targeted nuclear magnetic resonance (NMR) metabolomics approaches. Several metabolites correlated significantly to storage time and temperature. Citric acid decreased linearly over time at a temperature-dependent rate. Ethanol, formic acid, acetic acid, lactic acid, and succinic acid increased non-linearly after an initial period of minimal increase. Butyric acid exhibited strong inverse temperature dependencies. This study provides the first analysis of the effect of time and temperature on the concentration of key metabolites during milk storage. Candidate molecules for shelf-life monitoring have been identified, and the results improve our understanding of molecular changes during milk storage. These results will inform the development of real-time shelf-life indicators for milk, helping to reduce milk waste.

## 1. Introduction

Milk is a popular food consumed around the world. World milk production amounted to 839 billion kilograms in 2018 [1] and is estimated to reach 980 billion kilograms by 2028 [2]. The United States is among the largest producers of milk in the world, second only to India [3], producing 98.7 billion kilograms in 2018, with 21.6 billion kilograms sold as fluid milk products [4,5]. Overall, 2% fat milk accounted for 27% of liquid milk sales in the US in 2018, comparable to 33% from whole milk [5].

However, a significant portion of the milk produced is never consumed. In 2010, 32% of all liquid milk available at the retail level was wasted in the US, representing a loss of USD 6.4 billion and 10.8 billion calories from the food supply system [6]. This wasted food represents not just enormous resource losses but also contributes to detrimental environmental consequences after disposal. Date labels have been shown to contribute to consumer waste in a variety of food categories [7]. Milk date labels have been shown to increase discard intentions among consumers by up to 40% compared to containers without date labels [8]. Due to the static nature of date labels, they are unable to account for the effect of environmental factors such as temperature fluctuations during food storage and distribution. An improved solution to date labels could, therefore, reduce the amount of milk wasted. The development of this technology requires an understanding of the changes occurring during milk storage.

Milk shelf-life is primarily limited by the growth of Gram-negative psychrotrophic bacteria introduced during post-pasteurization contamination. A study investigating the bacterial ecology of milk from different parts of the United States observed *Pseudomonas* to be the predominant Gram-negative genus. Some Gram-positive bacteria such as *Bacillus*, *Paenibacillus* and some lactic acid bacteria have also been observed in milk [9]. However, *Pseudomonas* have been identified as the predominant post-pasteurization contaminants. In addition to bacterial contamination, heat-resistant lipases and proteases surviving pasteurization significantly affect off-flavor formation in milk and limit its shelf-life. Moreover, a strong correlation between volatile organic compounds generated in milk during storage and sensory acceptance has been previously observed [10]. Therefore, the end of the shelf life of milk should be considered the time when its sensory properties are no longer acceptable to consumers. The products of microbial metabolism are typically small organic molecules, many of which are volatile. Lactose is metabolized into acids, resulting in a sour flavor [11]. The combination of lower pH and bacterial proteases results in the coagulation and gelation of casein [12]. The degradation of proteins causes bitter off-flavors [13], while triglyceride breakdown results in off-flavors ranging from rancid to soapy to fruity [14,15]. Therefore, studying changes in milk metabolites could reveal potential indicators of spoilage. Since commercial HTST (high temperature, short time) pasteurization eliminates pathogenic microorganisms in raw milk, the compounds arising during pasteurized milk storage are due to the presence of non-pathogenic spoilage organisms and thermostable enzymes that survive pasteurization. While these compounds can be responsible for undesirable flavor and texture formation at the end of shelf life, they are unlikely to pose health risks.

Generally, a microbial count of 10^6^ to 10^7^ CFU/mL is used as an indicator of milk spoilage as populations of this size can produce off-flavors [16]. However, milk may still be considered acceptable by consumers at 10^6^ CFU/mL. The type of microorganisms and the amount of thermostable enzymes generated before pasteurization play a large role in the spoilage rate [17]. This makes shelf-life estimation difficult due to the microbial variability across batches and even within the same batch [18]. Further, the composition of the microbial population can influence the types and quantity of metabolites produced due to interactions between microbes [19].

Shelf-life indicators provide information regarding food quality by monitoring the internal or external environment of packaged foods [20] and are considered to mirror product quality more accurately than static date labels. Time–temperature indicators measure time and temperature to display a visual response, such as color change [21], and have been used in cold-chain operations where products are likely to experience higher than ideal refrigerated temperatures of 4–7 °C. Freshness indicators monitor compounds related to spoilage, such as ethylene, hydrogen sulfide [20], ethanol, or carbon dioxide [22]. Time–temperature indicators have been suggested for ultra-high temperature (UHT) treated milk [23] and pasteurized milk [24]. Ziyaina et al. (2019a) used a colorimetric sensor to detect volatile microbial products in pasteurized whole milk [25].

Several recent studies have been conducted to identify metabolite changes in pasteurized milk during storage. Alothman et al. (2018) analyzed pasteurized whole milk stored at 4.5 °C for up to 26 days using proton transfer reaction–mass spectrometry (PTR–MS) and tentatively identified 12 metabolites significantly changing over time [26]. Silcock et al. (2014) stored pasteurized milk of three fat contents (0.25–0.40%, 1.40–1.50%, and 3.18–3.28%) at 4.5 °C for up to 17 days and analyzed the samples with PTR–MS and solid phase micro extraction–gas chromatography–mass spectrometry (SPME–GC–MS) [27]. Differences between fat contents were observed, indicating a need to study milks with different fat percentages. Ziyaina et al. (2019b) analyzed pasteurized whole milk stored between 7 and 19 °C using SPME–GC. A number of metabolites changed during storage; however, these changes were not modeled [28]. Commercial pasteurized milk in the US is generally packaged in translucent monolayer HDPE containers. Karatapanis et al. (2006) studied the effect of different packaging materials on volatile metabolites generated in pasteurized milk during storage [29]. Among the five packaging materials studied, they concluded that multilayer pigmented HDPE was the best material for milk flavor retention, followed by paperboard cartons and monolayer pigmented HDPE containers. However, this study did not evaluate the combined effect of storage temperature and time on milk metabolites.

These studies provide evidence that metabolites can be used for shelf-life monitoring in milk. They also highlight a need for further experiments to study pasteurized milk of other fat percentages at different storage temperatures. However, these studies only examined volatile compounds, whereas microorganisms also produce non-volatile metabolites. Additionally, the quantification of compounds enables the modeling of concentration changes, which has not been attempted in recent metabolomics studies. Finally, to the best of our knowledge, there is no previous work looking at the combined effect of storage time and temperature on metabolites across milk sourced from multiple batches of pasteurization.

Nuclear magnetic resonance (NMR) can identify and quantify volatile and non-volatile molecules within mixtures of compounds and has been successfully used to analyze milk composition [30]. Hu et al. (2004) used NMR to characterize compounds of UHT whole milk, including fatty acids, citrate, and creatine [31]. Sundekilde et al. (2011) differentiated milk from different cow breeds [32], while Sacco et al. (2009) differentiated the geographic origins of milk via NMR [33]. Further, NMR has been used to identify mixtures of cow and sheep milk [34]. Belloque et al. (2001) used NMR to identify changes in phosphoglycerides during the storage of UHT milk at 10, 20, and 30 °C [35].

In the presented study, we use NMR spectroscopy to study volatile and non-volatile low-molecular-weight milk compounds in a controlled storage experiment. Our primary objective is to identify markers of milk spoilage in 2% fat milk using a metabolomics approach. Studying changes in milk metabolomic profiles during storage can provide insight into shelf life and inform the development of real-time shelf-life monitoring systems to reduce food waste.

## 2. Results and Discussion

### 2.1. Multivariate Analysis

**Figure 1:**
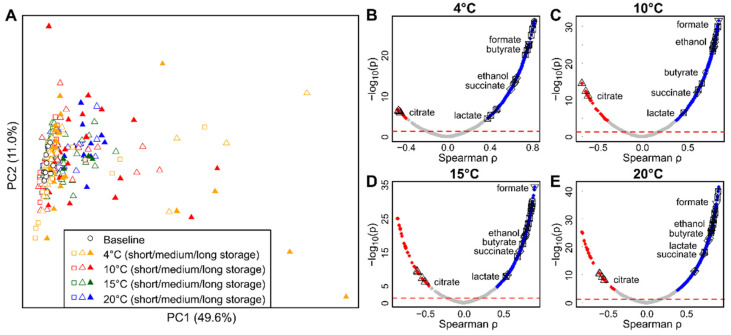
Untargeted NMR metabolomics analysis results. (**A**) Unsupervised analysis: principal component analysis of metabolite signals after collection (baseline) and during storage. (**B**–**E**) Supervised analysis: volcano plots of Spearman correlation analysis of metabolite signals and storage time at 4 (**B**), 10 (**C**), 15 (**D**), and 20 °C (**E**). Significant signals after FDR correction are highlighted in red (decreasing) or blue (increasing). The dashed line signifies nominal significance (*p* = 0.05). Identified metabolite signals are marked.

Representative NMR spectra of the analyzed milk samples are shown in Figures S1 and S2. Principal component analysis (PCA) was performed on the 1D NMR data to screen for strong group differences (Figure 1A). The PCA revealed strong effects of storage time on the metabolite profiles of the milk samples. Interestingly, the samples stored at different temperatures had different metabolic changes, indicated by different positions of the late storage samples in the PCA plot. Additionally, the PCA plot revealed that storage effects are not identical for all samples stored at the same temperature, probably owed to differences in initial microbial composition. To gain deeper insights into the change in metabolite levels, a supervised analysis was performed.

### 2.2. Supervised Untargeted Analysis

To identify metabolites that change during storage, Spearman correlation analysis was performed in a supervised, untargeted approach. This type of non-parametric analysis has the advantage that it can identify significant correlations even if their exact parameters (e.g., linear, exponential) are initially unknown. This analysis was performed for all observed signals in the NMR spectra, and the resulting *p*-values were corrected for multiple testing using FDR correction. A large number of signals were significantly correlated to storage time, indicating these molecules showed a significant consecutive increase or decrease in concentration over time. Out of 1050 total signals (bins), the number of significant signals after correction for multiple testing was 367, 440, 500, and 605 at 4, 10, 15, and 20 °C, respectively. This is visualized in Figure 1B–E as volcano plots of the correlation coefficient (Spearman ρ) versus the negative decadic logarithm. Signals that are both significant and impactful can be found toward the top left and top right in this kind of plot. From the plot, it is obvious that at all temperatures, there are more increasing than decreasing signals, with the lowest number of decreasing signals at 4 °C.

One goal of this study was to identify robust markers of milk spoilage; therefore, we focused on signals that exhibited significant change over time at all of the investigated storage temperatures. Signals that fulfilled these requirements were subjected to metabolite identification procedures. This led to the identification of the following metabolites: citric acid, ethanol, formic acid, acetic acid, lactic acid, succinic acid, and butyric acid. Volcano plots (Figure 1B–E) indicate that citric acid is the only metabolite that decreases during storage, while all other identified significant metabolites increase. It should be noted that although all of the identified metabolites are organic acids, the employed untargeted approach has the potential to identify metabolites from many other classes of chemical compounds. Finding a high number of organic acids indicates that this group of compounds is highly affected by milk spoilage in the analyzed milk type. A large number of other known milk metabolites were visible in the NMR spectra; however, in accordance with the goals of this study, we did not further investigate any metabolites that did not exhibit significant change during storage.

### 2.3. Targeted Analysis

**Figure 2:**
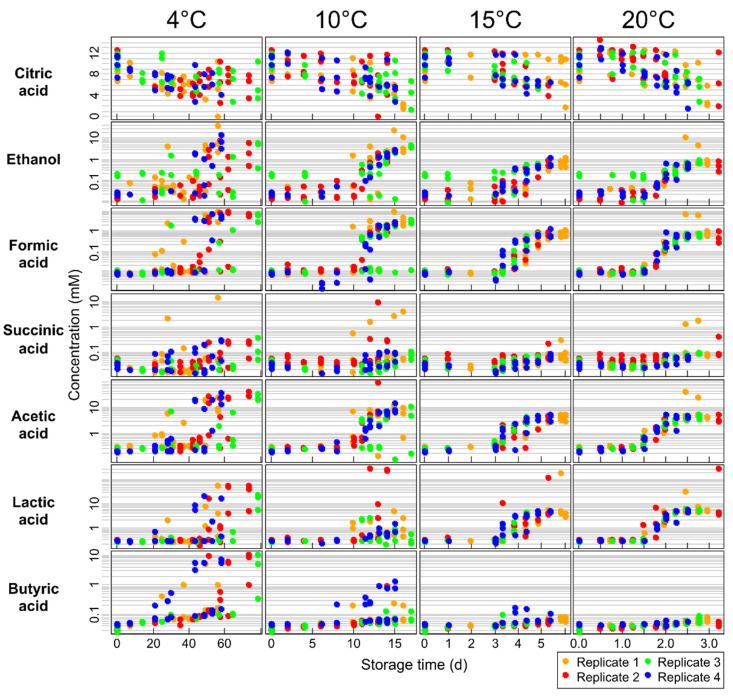
Targeted NMR metabolomics analysis results. Each row shows millimolar concentrations of one significantly changing metabolite versus storage time in days at 4 different storage temperatures. Citric acid uses a linear scale, while all other metabolites use a logarithmic scale on the *y*-axis (concentrations). The *y*-axes are identical for all plots in one row, while the *x*-axes are identical for all plots in one column.

The metabolites identified as being significant in the above untargeted analysis were then analyzed by a supervised, targeted approach to gain deeper insights into the nature of the observed change. Molar metabolite concentrations were quantified using ^1^H NMR and are shown in Figure 2. Quantitative results per sample can be found in Table S1. These concentration data were then analyzed using supervised multifactor analysis to investigate the relationships of storage temperature, storage time, milk batch, and their interaction terms on these metabolites. Multifactor ANOVA models identified multiple factors that significantly influenced metabolite concentrations during storage (Table 1).

### 2.4. Effect of Storage Temperature

Storage temperature had a significant effect on the concentrations of the selected target metabolites (Table 1). This was expected, as higher temperatures within the range used in this study are associated with higher microbial growth and elevated enzymatic activity. This also explains the significance of the interaction term between time and temperature in all metabolites, showing that the effect of storage time is different across temperatures. The square term of temperature had additional significant effects on all analyzed metabolites except for citric acid. This indicates the non-linear effects of storage temperature on the change in metabolite concentrations, as would be expected for metabolites affected by microbial growth that exhibits non-linear temperature dependence.

### 2.5. Effect of Milk Batch

Milk samples were collected from different batches of pasteurization, constituting biological replicates. Table 1 shows that the biological replicate caused significant changes in all metabolites. This is also obvious in Figure 2 by comparing different batches (replicates) at storage time 0. These inter-batch differences in baseline concentrations contribute to the relatively large interquartile ranges seen in Figure S3. It should be noted that this variability does not stem from technical imperfections but reflects actual biological differences between the different batches of milk. Reasons for batches differing in starting composition are various factors influencing milk composition, such as diet, lactation stage, breed, and season of milk collection.

Besides these batch differences, the interaction term between storage time and replicate was significant for the majority of metabolites. This indicates that metabolite concentrations change differently over time depending on the milk batch. This can be explained by the fact that each batch has a unique population of microorganisms that will affect metabolite levels differently depending on the microbes present in the respective batch. Assuming that metabolite concentrations are related to the rate of milk spoilage, this shows that different batches spoil at different rates.

These batch effects can be controlled for in a shelf-life indicator setting by analyzing metabolite change relative to the respective starting concentration compared to setting fixed threshold values. However, this control might not be necessary. Since consumer rejection is based on the sensing of microbial metabolites and not on sensing the actual microorganisms, a predefined threshold for a spoilage metabolite, regardless of initial concentration, is likely to correlate well with consumer rejection.

### 2.6. Citric Acid

Citric acid was the only milk metabolite that was significantly and consistently decreasing during storage time in this study. Figure 2 shows the effect of storage time on citric acid levels at all four storage temperatures. The concentration of citric acid decreases close to linearly over time. The variability in concentration data at each storage time is representative of the batch-to-batch variability present in the study.

The average initial concentration of citric acid in the 2% fat milk samples was 10.3 ± 0.4 mM. This is within the reported range of citric acid in milk of 3.03 to 12.9 mM [36,37]. Citric acid has been previously quantified in 2% fat milk at a level of 5.2 mM [38]. The concentration of citric acid in milk can vary due to seasonal changes and cow diet, with higher concentrations during the grazing season and as a result of high-fat diets [39]. The stage of lactation of the cow also has an impact on citric acid levels in milk, with the highest concentrations during the early stage of lactation [40,41]. It should be noted that despite the batch dependence on the initial concentration, citric acid changed consistently with time and temperature regardless of the milk batch. This is also indicated by the fact that the interaction terms time × replicate and temp × replicate were not significant (Table 1).

**Figure 3:**
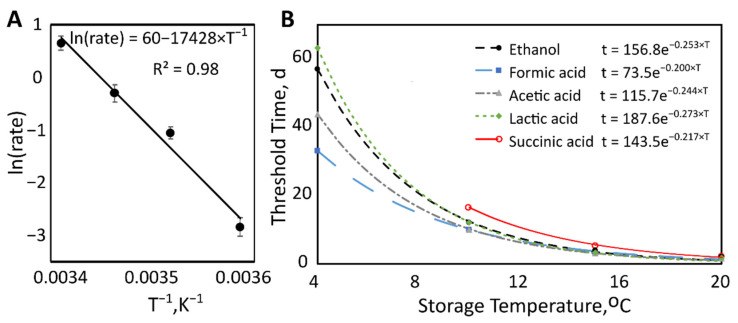
(**A**) Arrhenius plot showing the effect of temperature on the rate of citric acid degradation. Natural log of the rate with standard error bars by reciprocal absolute storage temperature (K^−1^). (**B**) Threshold time (days) by storage temperature (°C). Threshold time decreased exponentially as storage temperature increased.

Citric acid degradation rates were heavily temperature-dependent, with values of −0.06, −0.64, −0.73, and −1.91 mM/d for 4, 10, 15, and 20 °C, respectively. An Arrhenius plot was created using the natural log of the rate of citric acid degradation by the reciprocal absolute temperature (Figure 3A). The linearity of this plot provides evidence that the data fit the Arrhenius equation, describing the temperature dependence of this reaction rate. From this equation, the activation energy for the degradation of citric acid in milk was calculated to be 143 kJ/mol.

Citrate is the most abundant metabolite in milk behind lactose and other select inorganic ions and minerals [38]. Changes in citric acid concentration during milk storage have not been well studied, potentially due to a focus on volatile organic compounds in such studies. Bosworth and Prucha (1910) observed a considerable decrease in citric acid concentration during milk storage, although the details of their experiment are lacking [42]. A study on UHT milk found a slight increase in citric acid after storage for 90 days at ambient temperature, indicating that higher temperature treatment might reverse this effect [43]. The present study thus represents some of the first research attempts to quantify citric acid throughout milk storage. Citric acid is known to be fermented by some species of lactic acid bacteria [44,45,46]. However, the observed linear decrease does not fit well with a decrease purely resulting from microbial consumption as microbial growth exhibits strong non-linear patterns. Bacterial counts in pasteurized milk are very low directly after pasteurization and during the lag phase of microbial growth, initially leading to stable levels of citric acid, followed by an accelerated decrease once the number of bacteria is sufficient. This is contradicted by the observed linear decrease. Thermally resistant enzymes can, to some extent, survive the heat treatment of the pasteurization process, and the presence of a stable pool of enzymes would be expected to cause a linear decrease in citric acid. However, the activation energy of enzyme reactions is in the range of 0–35 kJ/mol, lower than the 143 kJ/mol calculated from the Arrhenius equation [47]. Further research is necessary to explain the mechanism of citric acid metabolism seen in this study. Despite the need for further analyses, we conclude that citric acid is a viable candidate for shelf-life monitoring in milk because of the well-defined effect of time and temperature.

### 2.7. Products of Lactose Fermentation

Ethanol, formic acid, acetic acid, and lactic acid changed significantly with storage time and the quadratic terms of time, indicating non-linear relationships (Table 1). The non-linear effect of storage time is evident in Figure 2.

The levels of these metabolites stayed relatively constant until a threshold time, after which the concentrations increased at an accelerated rate. This relates to microbial growth trends, which undergo a lag period of minimal growth, followed by an exponential increase. This is consistent with results from a study of whole milk stored at 4.5 °C, where concentrations of metabolites increased only after a threshold bacterial concentration of 10^6^–10^8^ CFU/mL was met [27].

Steel’s tests identified the earliest time points when metabolite concentrations significantly exceeded initial concentrations (Table 2). The average storage time at this collection time point was considered the “threshold time” for that temperature. For example, at 4 °C, the 10th collection time point had a significantly higher concentration of ethanol compared to time point zero. The average storage time corresponding to the 10th timepoint was then calculated to be 57 days. Plotting threshold time by storage temperature revealed exponential decreases of metabolite levels with increasing temperature (Figure 3B). The decay rates can be seen in Table 3.

#### 2.7.1. Ethanol

Ethanol increased non-linearly with storage time (Table 1, Figure 2). With an average initial concentration of 45 ± 61 µM, the high standard deviation was caused by two samples >150 µM, while the other samples were below 30 µM (Figure S3). Ethanol has been quantified in raw milk at 0.8 µM, but its concentration has been shown to vary depending on the cow’s diet [48].

Ziyaina et al. (2019) reported ethanol during the storage of pasteurized whole milk. However, the change in ethanol concentration was not reported at all storage temperatures [28]. Urbach and Milne (1987) identified increasing concentrations of ethanol during the storage of pasteurized milk at 4, 7, and 10 °C [49]. The production of ethanol began earlier at higher storage temperatures and increased with storage time and temperature, consistent with our findings. Ethanol is a product of lactose fermentation in many species of bacteria, including Enterobacteriaceae and *Lactococcus lactis* [50,51]. Pierami and Stevenson (1976) concluded that ethanol was an indicator of the growth of the psychotrophic bacteria *Pseudomonas fragi*, *Pseudomonas perolens*, and *Bacillus pumilus* in milk [52]. Haugen et al. (2006) found that ethanol was produced by two milk spoilage bacteria of the *Enterobacteriaceae* family, *Serratia marcescens* and *Serratia proteamaculans*, in milk [53].

#### 2.7.2. Acetic Acid

Acetic acid increased significantly in a non-linear way with storage time (Table 1, Figure 2). The average initial concentration of acetic acid was 91 ± 12 µM, and it has been reported at 30 µM in 2% fat milk [38].

Alothman et al. (2018) identified changes in acetic acid concentration at 4.5 °C [26], and Ziyaina et al. (2019b) discovered acetic acid generation in pasteurized whole milk [28]. Like ethanol, acetic acid is attributed to psychrotrophic bacteria [53], *S. marcescens*, *S. proteamacufans*, and *P. putida* [52]. Acetic acid can be produced by lipolysis, citrate fermentation, lactose fermentation, or amino acid metabolism [51,54,55].

#### 2.7.3. Formic Acid

Formic acid increased in a non-linear way with storage time (Table 1, Figure 2). Formic acid was found at an initial concentration of 8.3 ± 1.3 µM, and it has been reported at 16 µM in 2% fat milk [38].

Formic acid has been reported less frequently than ethanol and acetic acid. Harper, Gould, and Hankinson (1961) found increasing levels of formic acid over time during the storage of pasteurized milk at 4.4 °C [56]. Formic acid is produced by *Enterobacteriaceae* and several species of lactic acid bacteria [57]. Formic acid is a product of lactose fermentation in dairy starter cultures, along with ethanol, acetic acid, and the odor active compounds diacetyl, acetoin, and 2,3-butanediol [58]. Formic acid is also produced during citrate fermentation in lactic acid bacteria species [44,59].

#### 2.7.4. Lactic Acid

Lactic acid increased in a non-linear way with storage time (Table 1, Figure 2). The average initial concentration of lactic acid was 87 ± 5 µM. Previously, lactic acid has been quantified in 2% fat milk at 57 µM [38].

Lactic acid is produced as a major end product of lactose and citrate metabolism in Enterobacteriaceae and lactic acid bacteria in milk [44]. Although the presence of this metabolite in pasteurized milk has been well documented, no storage studies that have examined its concentration over time and temperature were found. Therefore, this study is the first to quantify changes in lactic acid concentration in pasteurized milk as a function of time and temperature.

Overall, ethanol, formic acid, acetic acid, and lactic acid are all appropriate candidates for shelf-life monitoring in pasteurized 2% fat milk. The effect of time and temperature is well defined for each metabolite, making the prediction and modeling of the changes in concentration feasible. Further research is needed to confirm the results of this study, accounting for seasonal and regional differences.

### 2.8. Succinic Acid

Succinic acid increased significantly and non-linearly with time (Table 1, Figure 2). Succinic acid concentration increases over time, although the magnitude of the change is lower than for ethanol, formic, acetic, and lactic acid. The concentration, again, stays relatively consistent for a period of time, but the threshold time after which the concentration increases is less apparent visually compared to the other metabolites discussed previously.

Initial concentrations of succinic acid were at 41 ± 10 µM. Succinic acid has been previously quantified at 15 µM in 2% fat milk [38]. The metabolite has been found in milk at levels up to 106 µM [36].

Succinic acid threshold times, at which the concentration significantly exceeded the initial concentrations, were greater than those of ethanol, formic acid, acetic acid, and lactic acid (Table 2). At 4 °C, the change in succinic acid levels was minimal, and the threshold time could not be determined throughout the course of this study.

As storage temperature increased, the threshold time decreased exponentially, following the same trend as ethanol, formic acid, acetic acid, and lactic acid (Figure 3B). The confidence interval overlapped with the decay rates for ethanol, formic acid, and acetic acid, but not lactic acid (Table 3).

Succinic acid is produced by *Enterobacteriaceae*, *Bacillus* species, many strains of *Lactobacillus*, and other lactic acid bacteria species [45,57]. This organic acid is produced as an intermediate product in the Krebs cycle and is an end product of citric acid fermentation. The amount of succinic acid produced is dependent on the lactic acid bacteria strains present, pH, and temperature [57]. Succinic acid contributes to the flavor of fermented dairy products, which may not be desirable in milk [45].

This study provides the first analysis of succinic acid concentration during milk storage. Although succinic acid has been previously detected in pasteurized milk, the changes in concentration of this metabolite have not been explored in other storage studies. The temperature and time dependence of succinic acid concentration indicates this compound could be useful in the shelf-life monitoring of milk.

### 2.9. Free Butyric Acid

Figure 2 shows free butyric acid levels increasing during storage at 4 °C. The level of free butyric acid initially present in the milk samples was 37.2 ± 0.7 µM, in accordance with Foroutan et al. (2019), who reported a concentration of 37 µM in 2% fat milk [38].

Butyric acid exhibited highly unusual behavior. At 20 °C, butyric acid showed a slow increase over time. However, at lower temperatures, a stunning concentration increase was visible in a subset of samples, with the highest levels reached at 4 °C.

Butyric acid has been identified in milk storage studies as a product of bacterial metabolism. Silcock et al. (2014) found butyric acid in milk stored at 4.5 °C at increasing concentrations over time using PTR–MS [27]. Ziyaina et al. (2019b) detected butyric acid in 3.9% fat milk between 7 and 19 °C using GC–MS, with levels frequently below the detection limit [28]. The concentrations of butyric acid in the study by Ziyaina et al. (2019b) were difficult to predict based on temperature and time, similar to the results of our study [28].

Butyric acid is a short-chain fatty acid that, in low concentrations, produces a cheesy odor in milk [26]. In a study of milk from Sweden, butyric acid comprised 4.4% by weight of total free fatty acids [60]. The metabolite can be detected in milk after lipolysis occurs [61]. Özcelik et al. (2016) identified butyric acid as a product of lactic acid bacteria metabolism [57]. Butyric acid can also be formed by thermostable lipases of psychrotrophic microbes that are present in milk before pasteurization [62,63]. At the same time, butyric acid may be consumed by various microbes, although research on this topic is scarce. Belenguer et al. (2006) specifically identified the butyrate-consuming microbe *Bifidobacterium adolescentis* [63]. The genera *Bifidobacterium* is common in cow’s milk [64], and *B. adolescentis* has been identified in human milk and bovine rumen [65], making it possible that this species may be present in cow’s milk as well. Although *Bifidobacteria* are inactivated through pasteurization, we hypothesize that the drop in butyric acid levels observed at higher temperatures may be caused by the increased activity of butyric-acid-consuming microbes that are less active at lower temperatures.

In this study, the observed levels of butyric acid were subject to multiple issues. We conclude that butyric acid is not a good candidate for monitoring shelf life in 2% fat pasteurized milk. However, due to its high sensitivity to elevated temperatures, this metabolite might be used to detect temperature abuse during milk storage.

## 3. Materials and Methods

### 3.1. Sample Collection and Preparation

**Figure 4:**
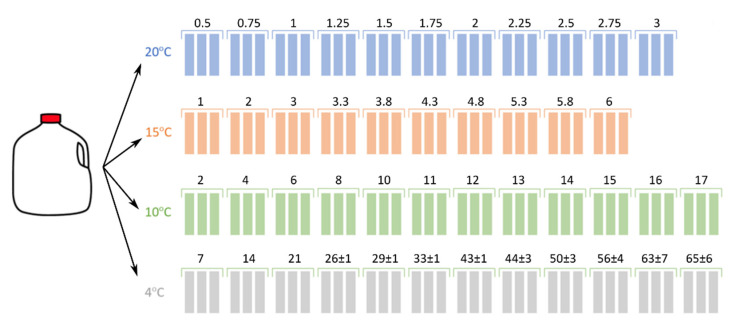
Sample collection times (days) for each storage temperature. Shown are means ± standard errors (omitted if equal to zero).

2% fat HTST (high temperature, short time) pasteurized bovine milk in one gallon (3.78 L) milk containers were picked up on the day of pasteurization from Tamarack Farms Dairy (Newark, OH, USA). These milk containers were then transported in ice-chests (temperature below 4 °C for 40 min) to the Ohio State University Food Science building. This was repeated once per week for four consecutive weeks between June and July 2019 in order to collect biological replicates for analysis. In total, 4 mL of milk from each milk container was saved as a control (time point zero) sample and stored at −80 °C until further analysis. The remaining milk was transferred to sterile glass bottles and stored at 4, 10, 15, and 20 °C in the dark. Samples were collected over a time period of 3, 6, 17, and 78 days for 20, 15, 10, and 4 °C, respectively. Collection time points and sample sizes were chosen based on preliminary results from previous experiments. At 4 °C, the later collection intervals were adjusted based on the perceived rate of off-odor production and texture. The collection regime is shown in Figure 4. For the purpose of group assignment blinding, bottles were only marked to indicate the replicate number (pick-up date) but were not further labeled, e.g., to assign storage times. At each collection time, three bottles were randomly selected from all bottles of this replicate number and removed from storage. Samples that were visibly spoiled were excluded from analysis; this was mostly the case for late-stage samples from storage at 20 °C.

Samples were prepared as described by Klein et al. (2010) [36]. The samples were removed from storage and thawed in a water bath, then vortexed to achieve homogeneity. The milk was ultra-filtered using Amicon Ultra-2 centrifugal filter units (EMD Millipore Corporation, Burlington, MA, USA) with a molecular weight cutoff of 10 kDa. This filtering step removes macromolecules such as proteins and lipids and larger particles that would impede NMR spectral analysis. These regenerated cellulose filters filter by particle size, not by molecular weight, and the cutoff of 10 kDa is the estimated molecular weight for the threshold particle size. Of note, this cutoff is not a hard threshold, meaning that even smaller macromolecules will be retained by the filters to some extent. It should be noted that some small milk proteins/enzymes might escape the filters, potentially posing issues during data analysis. For the metabolites and concentration ranges observed by NMR, this seems to be a minor issue, as previous studies have successfully used this sample preparation method [36]. However, for platforms such as liquid chromatography-mass spectrometry, other methods such as methanol precipitation may be advantageous. After filtration, deuterium oxide (D2O) containing 3-trimethylsilyl-2,2,3,3-tetradeuteropropionate (TSP) as an internal standard and boric acid to prevent bacterial growth was added, and the pH of each sample was adjusted to 7.4.

### 3.2. Nuclear Magnetic Resonance Spectroscopy

NMR experiments were conducted on a Bruker Avance III HD Ascend 850 MHz spectrometer (Bruker BioSpin, Rheinstetten, Germany) with a triple-resonance inverse cryoprobe, z-gradients, and an automatic sample changer, as described earlier [36]. The probe was automatically locked, tuned, matched, and shimmed for each sample. One-dimensional (1D) ^1^H nuclear Overhauser enhancement spectroscopy (NOESY) spectra were collected at 298 K. Two samples were analyzed via ^1^H-^13^C heteronuclear single quantum coherence (HSQC) NMR to aid metabolite identification. Spectra were automatically Fourier-transformed and manually phase-corrected using TopSpin 3.6.2 (Bruker BioSpin). A line broadening of 0.3 Hz and an automatic baseline correction were applied. NMR spectra were analyzed by binning with a bin width of 0.01 ppm in AMIX 3.9.15 (Bruker BioSpin). NMR spectra that did not meet quality criteria for peak shape and linewidth were excluded from the analysis. This left a total of 374 spectra for analysis in this study.

### 3.3. Data Analysis

Statistical analysis of bin data was carried out in R version 3.6.2 (The R Foundation for Statistical Computing, Vienna, Austria). Spearman’s rank correlation coefficients and corresponding *p*-values were calculated for each bin over time at a given temperature. The *p*-values were corrected for multiple testing by controlling the false discovery rate (FDR) at the 0.01% level [66]. Significant peaks were identified by comparison with the Milk Composition Database (MCDB, http://mcdb.ca/, accessed on: 11 March 2020) and the Biological Magnetic Resonance Data Bank (BMRB, http://bmrb.wisc.edu/, accessed on: 11 March 2020). These significant metabolites were then quantified from 1D spectra using AMIX and MetaboQuant 1.3 [67]. Briefly, for each metabolite, a list of available peaks, including chemical shift, number of contributing protons, multiplicity patterns, and coupling constants, were manually entered into a “knowledge base” text file. AMIX uses this information to automatically fit the expected line shape and, if successful, calculate the peak areas. MetaboQuant then applies quality control algorithms and calibration factors to calculate absolute concentration values. Peaks were calibrated using standard solutions of pure compounds. Metabolite concentration data were analyzed in JMP 14 (SAS Institute, Cary, NC, USA). Multifactor analyses of variance (ANOVA) models were created to include storage time, storage temperature, biological replicate, square terms of time × time, temperature × temperature, and interaction terms time × temperature, time × replicate, and temperature × replicate. Storage time and storage temperature were treated as continuous variables, while biological replicate was discrete. Insignificant terms were removed one by one until all remaining terms were significant (*p* < 0.05). Homogeneity of variances, normality, and independence were tested, and if the assumptions were violated, a transformation was used to alleviate these issues. The data in this study required either a SHASH transform or a Johnson transform. Nonparametric Kruskal–Wallis tests were used to confirm the effect of time point on metabolite concentration at each storage temperature. Post-hoc non-parametric means comparisons were completed using Steel’s test to compare mean metabolite concentrations at each time point with the mean concentration present in the milk samples at time point zero. This test was used to identify the first collection time point when metabolite concentration was significantly higher than the initial concentration. The average storage time at this time point is referred to as the “threshold time” in this study. Decay rates were calculated using an exponential model of threshold time by storage temperature.

The focus of this study was monitoring and modeling changes in metabolite concentrations during milk storage using metabolomics. While this study successfully measured changes in metabolites as they relate to storage time and temperature, other factors such as feed, season, efficacy of pasteurization, and level of post-pasteurization contamination may affect the rates of milk spoilage. Further study is required to understand if certain metabolites are existent irrespective of factors such as feed, seasonality, or processing steps, including fat percentage. Such molecules may be best suited for shelf-life monitoring. Further, understanding how consumer end-of-shelf-life correlates with metabolite changes can provide an improved representation of milk shelf life and discard.

## 4. Conclusions

This study was the first to investigate changes occurring in milk as a function of storage temperature, time, and batch of pasteurization using untargeted and targeted NMR metabolomics. This led to the identification and quantification of several volatile and non-volatile organic compounds that changed as a function of storage temperature and time. Multiple metabolites changed significantly with storage time and temperature in a predictable manner. Citric acid decreased linearly over time at a rate dependent on temperature. Ethanol, formic acid, acetic acid, lactic acid, and succinic acid had a period of no increase, followed by a rapid increase after a threshold time. This threshold time decreased exponentially as storage temperature increased. The production of butyric acid during milk storage was highly temperature-dependent, appearing more frequently and at higher concentrations at lower temperatures. Butyric acid might be a candidate for detecting temperature abuse. The results from this study provide the basis for improved shelf-life prediction and monitoring in pasteurized milk, which will ultimately reduce the amount of milk wasted. Further research will be necessary to assess the generalizability of these findings regarding potential confounding influences on these milk metabolites.

## Figures and Tables

**Table 1:** ANOVA significance table for the effects of storage time, storage temperature, and biological replicate (batch) and their interaction and square terms on metabolite concentrations. Shown are *p*-values.

Effect	Citric Acid	Ethanol	Formic Acid	Acetic Acid	Lactic Acid	Succinic Acid	Butyric Acid
Storage Time	<0.0001	<0.0001	<0.0001	<0.0001	<0.0001	<0.0001	<0.0001
Temperature	<0.0001	<0.0001	<0.0001	<0.0001	<0.0001	<0.0001	<0.0001
Replicate	0.0031	<0.0001	0.0144	0.0002	<0.0001	<0.0001	<0.0001
Time × Time	0.0001	<0.0001	<0.0001	<0.0001	<0.0001	<0.0001	0.0793
Temp × Temp	0.0514	0.0007	<0.0001	<0.0001	0.0001	0.0007	0.0008
Time × Temp	<0.0001	<0.0001	<0.0001	<0.0001	0.0007	<0.0001	<0.0001
Time × Replicate	0.5097	<0.0001	<0.0001	<0.0001	<0.0001	<0.0001	0.0626
Temp × Replicate	0.2335	0.4694	0.0269	0.0674	0.5476	0.7023	0.1943

**Table 2:** Time until a significant change in metabolite concentration was observed (“threshold time”). Shown is mean ± standard deviation of threshold time points and *p*-value of comparison between the concentration at the threshold time point and the initial concentration at each storage temperature.

Metabolite	Temperature (°C)	Threshold Time (d)	*p*-Value
Ethanol	4	57 ± 7	0.035
	10	12.01 ± 0.01	0.043
	15	3.82 ± 0.01	0.037
	20	1.98 ± 0.02	0.006
Formic acid	4	33 ± 2	0.045
	10	9.97 ± 0.01	0.021
	15	3.31 ± 0.01	0.007
	20	1.78 ± 0.01	0.008
Acetic acid	4	44 ± 5	0.027
	10	9.97 ± 0.01	0.008
	15	3.00 ± 0.03	0.023
	20	1.49 ± 0.02	0.002
Lactic acid	4	63 ± 12	0.035
	10	12.01 ± 0.01	0.043
	15	3.31 ± 0.01	0.037
	20	1.78 ± 0.01	0.006
Succinic acid	4	-	-
	10	16.5 ± 0.5	0.021
	15	5.33 ± 0.04	0.006
	20	2.23 ± 0.01	0.009

**Table 3:** Decay rate parameter with a 95% confidence interval from the exponential function of threshold time by storage temperature.

Metabolite	Decay Rate (°C^−1^)	95% Confidence Interval
Ethanol	−0.254	−0.273 to −0.235
Formic Acid	−0.200	−0.211 to −0.190
Acetic Acid	−0.244	−0.256 to −0.231
Lactic Acid	−0.273	−0.290 to −0.255
Succinic Acid	−0.217	−0.243 to −0.191

## Data Availability

The data presented in this study are available in the supplementary material (concentration data) and at https://doi.org/10.5281/zenodo.5030794 (bin data), accessed on: 11 March 2020.

## Supplementary Materials

The following are available online at https://www.mdpi.com/article/10.3390/metabo11070419/s1, Figure S1: Representative one dimensional (1D) 1H NOESY spectrum of a milk sample, Figure S2: Representative two dimensional (2D) 1H-13C HSQC spectrum of a milk sample, Figure S3: Boxplots of initial metabolite concentrations (mM), Table S1: Replicate, storage temperature (°C), collection time point, storage time (h), and concentrations of all quantified metabolites (mM).
