# Supplementary material for: Metabolomic Markers of Storage Temperature and Time in Pasteurized Milk
Source: Metabolites. 2021 Jun 25;11(7):419. doi: 10.3390/metabo11070419 (PMC8306400; doi:10.3390/metabo11070419)
Supplement: Supplementary file 1 [file metabolites-11-00419-s001.zip › metabolites-1268976-supplementary.pdf]

Supplemental Material

Metabolomic Markers of Storage Temperature and Time in Pasteurized Milk

Kara M. Edwards[1], Aishwarya Badiger[1], Dennis R. Heldman[1], Matthias S. Klein[1]

[1]Department of Food Science and Technology, The Ohio State University, Columbus, Ohio, USA

Supplemental Figures

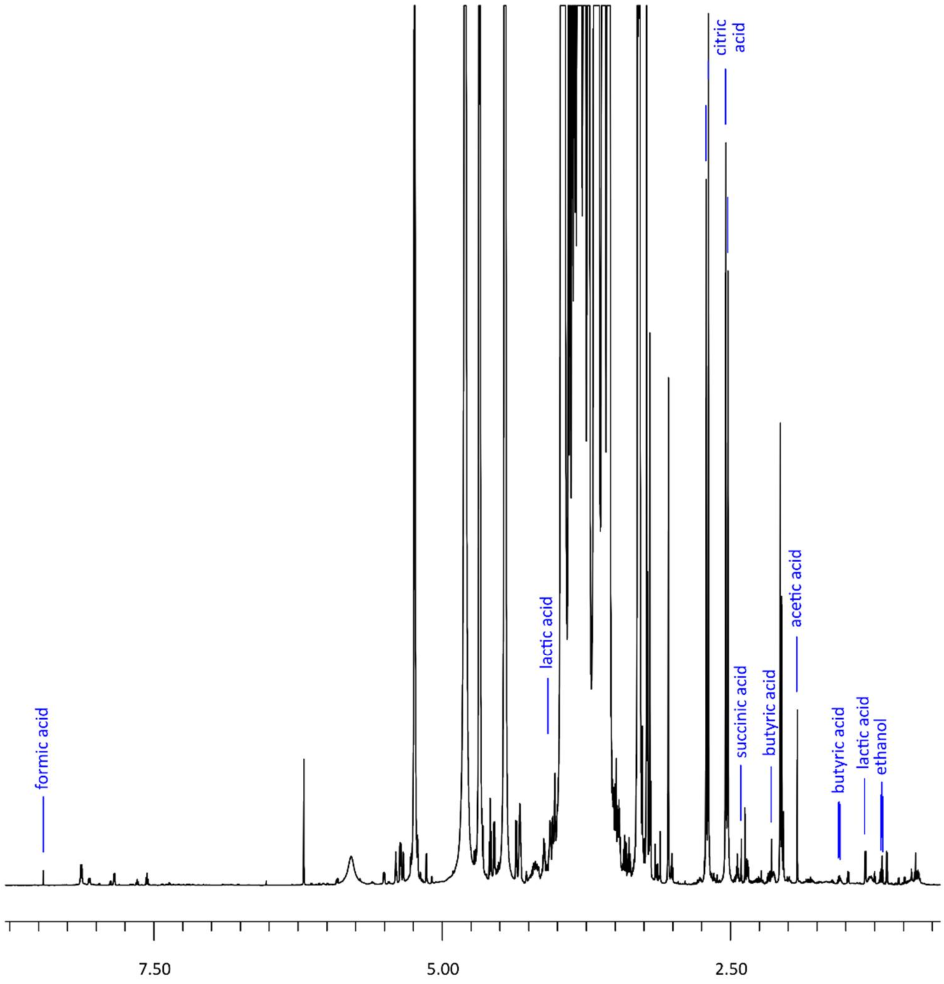

Figure S1. Representative one dimensional (1D) ^{1}H NOESY spectrum of a milk sample.

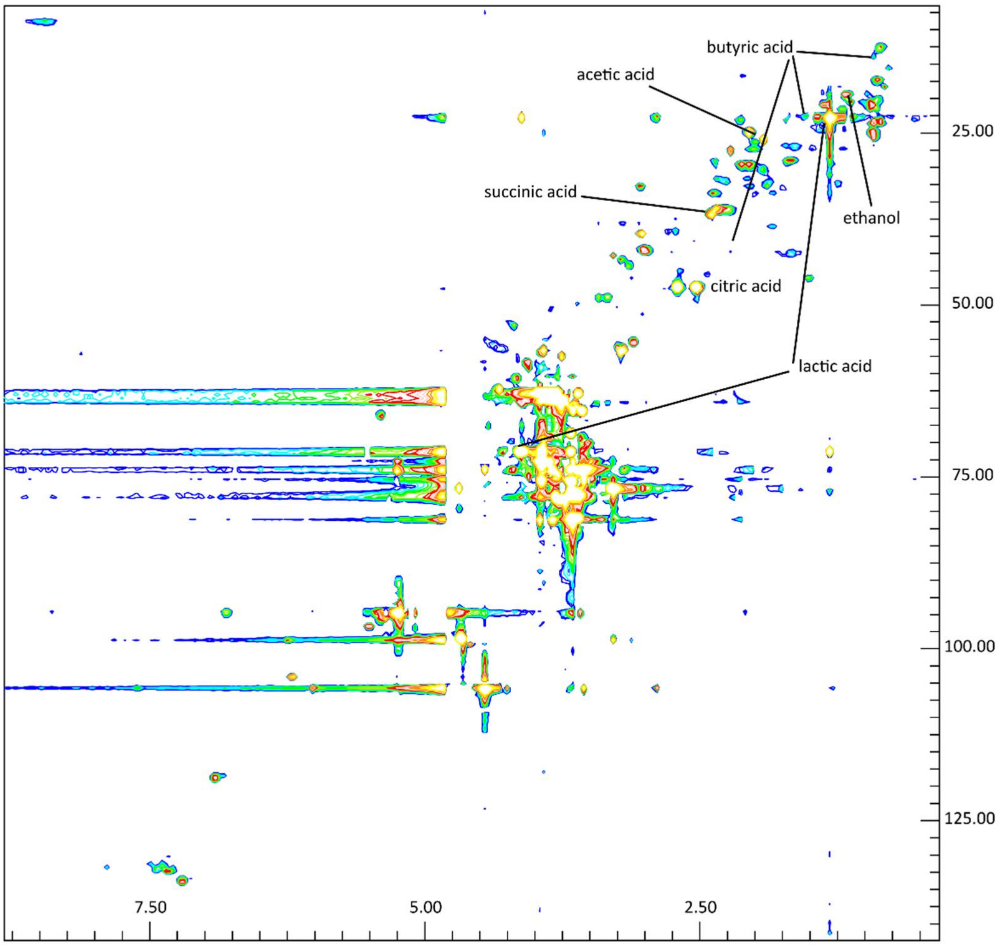

Figure S2. Representative two-dimensional (2D) ^{1}H-^{13}C HSQC spectrum of a milk sample.

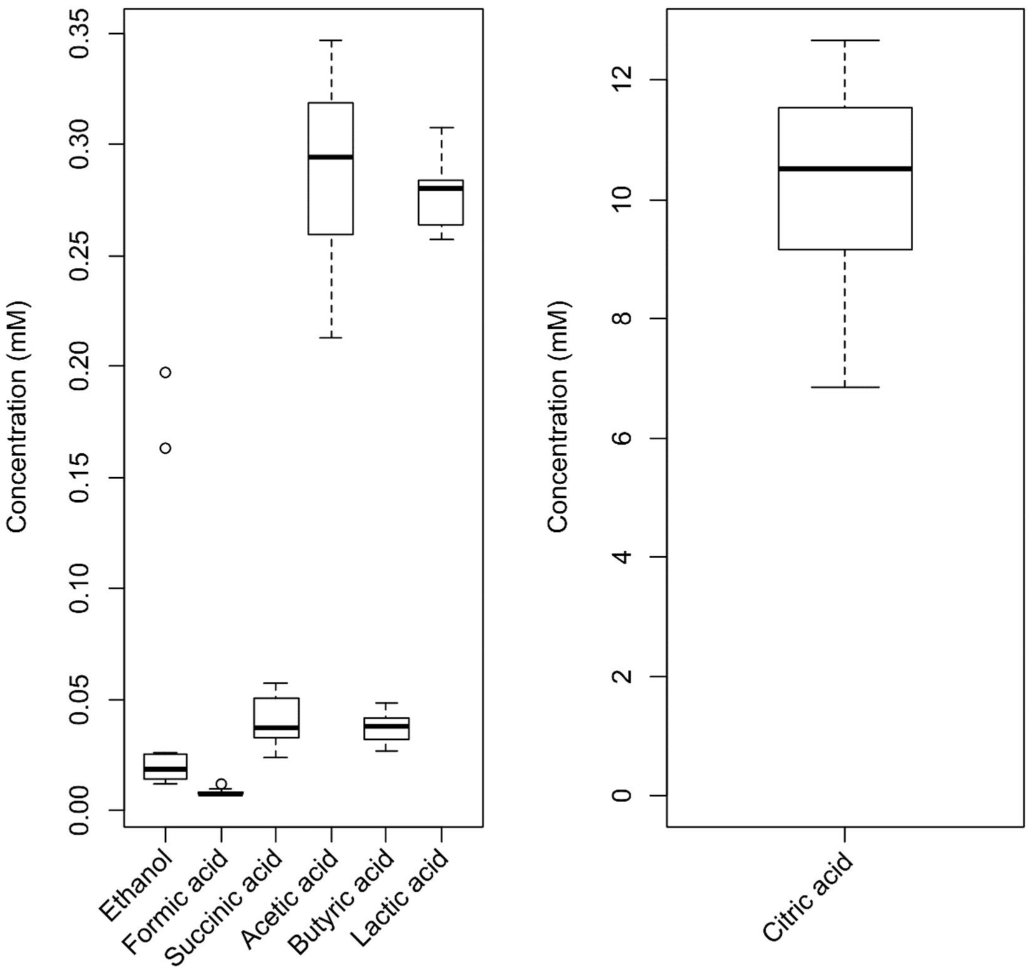

Figure S3. Boxplots of initial metabolite concentrations (mM).

Supplemental Tables

Table S1. Replicate, storage temperature (^oC), collection time point, storage time (h), and concentrations of all quantified metabolites (mM). NA=not available.

Replicate	Temperature (°C)	Time point	Storage time (h)	Ethanol	Formic acid	Succinic acid	Citric acid	Acetic acid	Butyric acid	Lactic acid
1	-	0	0	0.0125	0.0069	0.0286	6.8379	0.3359	0.0400	0.2611
1	-	0	0	0.0141	0.0088	0.0336	8.4775	0.3366	0.0356	0.2572
1	-	0	0	0.0200	0.0099	0.0389	9.9290	0.3470	0.0383	0.2605
2	-	0	0	0.0143	0.0073	0.0463	NA	0.2666	0.0314	0.2796
2	-	0	0	0.0132	0.0081	0.0521	11.5434	0.2770	0.0332	0.2941
2	-	0	0	NA	0.0077	0.0559	11.4377	0.2941	0.0292	0.2974
2	-	0	0	0.0146	0.0094	0.0577	12.6869	0.3004	0.0269	0.3075
3	-	0	0	0.1632	0.0123	0.0327	9.1708	0.2916	0.0272	0.2591
3	-	0	0	0.1971	0.0080	0.0362	12.0551	0.3095	0.0344	0.2818
3	-	0	0	NA	0.0081	0.0499	11.1083	0.3232	0.0383	0.2828
3	-	0	0	NA	0.0080	0.0526	9.5597	0.3144	0.0394	0.2799
4	-	0	0	0.0246	0.0068	0.0239	7.3843	0.2127	0.0436	0.2663
4	-	0	0	0.0260	0.0084	0.0299	9.7955	0.2410	0.0483	0.2789
4	-	0	0	0.0186	0.0076	0.0377	11.5877	0.2460	0.0447	0.2822
4	-	0	0	0.0194	0.0076	0.0370	11.3724	0.2522	0.0474	0.2846
1	4	1	169	0.0726	0.0076	0.0226	10.3611	0.3334	0.0438	0.2652
1	4	1	169	NA	0.0089	0.0234	7.7470	0.3721	0.0407	0.2744
1	4	1	169	0.0762	0.0082	0.0253	10.2273	0.3865	0.0436	0.2896
4	4	1	168.1	NA	0.0076	0.0202	9.2665	0.2530	0.0520	0.2743
4	4	1	168.1	0.0220	0.0070	0.0210	8.7707	0.2433	0.0565	0.2675
4	4	1	168.1	0.0222	0.0068	0.0213	8.7438	0.2519	0.0508	0.2603
3	4	2	336.7	0.2180	0.0092	0.0188	6.5207	0.3102	0.0571	0.2631
3	4	2	336.7	0.2408	0.0068	0.0182	7.0499	0.2905	0.0522	0.2532
3	4	2	336.7	0.0117	0.0088	0.0222	8.3294	0.3127	0.0550	0.2794
1	4	3	504.2	0.0491	0.0070	0.0163	5.8089	0.3309	0.0536	0.2592
1	4	3	504.2	0.0177	0.0109	0.0243	8.0959	0.4120	0.0763	0.3111
1	4	3	504.2	0.1363	0.0737	0.0434	8.1474	0.9395	0.0619	0.4130
2	4	3	503.6	0.0159	0.0089	0.0306	5.7436	0.3020	0.0520	0.2643
2	4	3	503.6	0.0385	0.0105	0.0366	5.5643	0.3115	0.0526	0.2635
2	4	3	503.6	0.0349	0.0102	0.0229	NA	0.2886	0.0569	0.2918
4	4	3	504.2	NA	0.0093	0.0217	7.8882	0.2473	0.0720	0.2727
4	4	3	504.2	0.0286	0.0078	0.0210	8.2224	0.2571	0.0775	0.2635
4	4	3	504.2	NA	NA	0.0921	5.1242	0.3965	0.2142	0.6691
1	4	4	600	0.0521	0.0085	0.0189	8.3990	0.3668	0.0600	0.2656
1	4	4	600	0.1002	0.1037	0.0463	5.8215	1.0902	0.0567	0.3684
1	4	4	600	NA	0.0130	0.0873	5.3257	0.9720	0.4086	NA
3	4	4	600.5	0.0331	0.0098	0.0176	12.0599	0.3157	0.0607	0.2876
3	4	4	600.5	0.2472	0.0085	0.0162	6.5475	0.3105	0.0589	0.2726
3	4	4	600.5	0.0243	0.0093	0.0173	11.1110	0.3107	0.0574	0.2736
4	4	4	672.2	NA	0.0090	0.0216	7.9043	0.2462	0.0736	0.2685
4	4	4	672.2	NA	NA	0.0444	5.3997	0.4978	0.2980	NA
4	4	4	672.2	NA	0.0110	0.0764	7.2167	0.5574	0.2953	NA
1	4	5	671.1	0.0478	0.0085	0.0162	5.4335	0.3637	0.0716	0.2616
1	4	5	671.1	NA	0.0088	0.0178	7.0570	0.3655	0.0739	0.2709
1	4	5	671.1	4.1550	2.6361	2.4362	4.3598	6.1786	0.0799	2.1453
3	4	5	720.2	0.1891	0.0085	0.0229	6.6105	0.4347	0.0843	0.2599
3	4	5	720.2	1.4866	2.0202	0.0471	6.9155	7.2239	0.1052	0.3927
4	4	5	720.5	NA	0.0077	0.0194	7.6546	0.2483	0.0843	0.2728
4	4	5	720.5	NA	0.0075	0.1123	NA	0.6767	0.5535	NA
4	4	5	720.5	0.0291	0.0098	0.0189	5.2982	0.2685	0.0840	0.2652

1	4	6	743.5	0.0589	0.0186	0.0169	5.9602	0.5063	0.0759	0.2692
1	4	6	743.5	0.0506	0.0120	0.0217	7.3314	0.5085	0.0747	0.2828
1	4	6	743.5	0.0272	0.0090	0.0168	6.0774	0.3591	0.0720	0.2414
2	4	6	839.7	0.0196	0.0137	0.0159	5.1773	0.2327	0.0460	0.2973
2	4	6	839.7	0.0176	0.0092	0.0282	3.9289	0.3262	0.0724	0.2669
2	4	6	839.7	0.0603	0.0142	0.0421	7.0415	0.4100	0.0704	0.3182
2	4	7	1007	0.0160	0.0163	0.0411	4.2817	0.6471	0.0730	0.3145
2	4	7	1007	0.0135	0.0094	0.0256	3.9392	0.3272	0.0727	0.2651
2	4	7	1007	0.0991	0.0126	0.0382	6.2997	0.4487	0.0678	0.3116
3	4	7	1056	0.1993	0.0102	0.0168	4.7745	0.3088	0.0779	0.2600
3	4	7	1056	0.2501	0.0086	0.0159	7.6342	0.3274	0.0857	0.2568
3	4	7	1056	NA	0.0113	0.0321	7.5862	0.3765	0.0837	0.2742
4	4	7	1034	1.8102	3.8219	0.1508	9.9242	17.6027	3.2989	5.9651
4	4	7	1034	0.0231	0.0091	0.0158	5.6140	0.2575	0.0924	0.2798
4	4	7	1034	1.9824	4.4327	0.0991	2.9031	18.5298	5.8753	8.5462
1	4	8	886.5	0.0126	0.0100	0.0208	7.6907	0.3977	0.0825	0.2773
1	4	8	886.5	NA	0.3020	0.1596	6.1402	2.6663	1.0869	NA
1	4	8	886.5	0.0269	0.0099	0.0175	4.9091	0.3709	0.0824	0.2772
2	4	8	1103.8	0.0507	0.0643	0.0492	7.9967	0.8020	0.0910	0.2173
2	4	8	1103.8	0.0183	0.0273	0.0285	4.0815	0.6302	0.0817	0.1780
2	4	8	1103.8	0.0368	0.0128	0.0313	6.8583	0.5256	0.0868	0.3512
4	4	8	1154	0.0181	0.0096	0.0201	8.0691	0.2513	0.0936	0.2661
4	4	8	1154	1.0874	3.4724	0.2037	NA	9.2745	5.5010	21.2601
4	4	8	1154	0.0588	0.0119	0.0152	5.6192	0.3303	0.1008	0.2757
1	4	9	960	NA	0.0097	0.0201	6.6383	0.3813	0.0710	0.2772
1	4	9	960	0.1325	0.0096	0.0157	6.1668	0.3579	0.0761	0.2617
2	4	9	1224	3.1025	4.6288	0.0661	8.9307	14.7430	0.1307	0.8553
2	4	9	1224	0.0624	0.1241	0.0494	5.6936	0.9308	0.1452	0.3175
2	4	9	1224	1.4364	3.0297	0.2624	7.4086	26.2802	9.7440	18.5383
3	4	9	1367.5	NA	0.0111	0.0198	8.2053	0.4016	0.1012	0.2817
3	4	9	1367.5	0.0244	0.0108	0.0210	8.9028	0.3222	0.0979	0.2727
3	4	9	1367.5	0.1911	0.2666	0.0395	6.3227	2.1045	0.1044	0.3399
4	4	9	1273.6	0.5014	0.3340	0.0257	6.1080	1.4413	0.1103	0.2939
4	4	9	1273.6	5.1339	7.4417	0.0721	8.2568	19.8250	0.1503	1.9116
1	4	10	1055.8	NA	0.0095	0.0196	7.4109	0.3299	0.0704	0.2521
1	4	10	1055.8	0.1373	0.0088	0.0157	6.2041	0.3167	0.0680	0.2754
1	4	10	1055.8	0.0303	0.0109	0.0173	5.2104	0.3260	0.0771	0.2733
2	4	10	1369.5	8.3175	8.4152	0.1024	10.4863	21.0472	0.3169	1.1785
2	4	10	1369.5	0.0223	0.0098	0.0197	6.4999	0.3245	0.0880	0.2633
2	4	10	1369.5	0.1450	0.3102	0.0575	2.7117	4.3109	0.5897	0.9740
3	4	10	1537.8	1.3254	1.2000	0.0326	2.9599	6.4540	0.1132	0.7054
3	4	10	1537.8	0.0282	0.0115	0.0228	8.7863	0.6228	0.1000	0.2770
3	4	10	1537.8	0.0169	0.0134	0.0299	7.2945	0.3836	0.1056	0.2678
4	4	10	1393.3	15.7957	5.9601	0.3356	NA	34.7287	5.9170	NA
4	4	10	1393.3	3.1723	4.0218	0.0481	4.1279	12.7411	0.1522	0.4163
4	4	10	1393.3	7.1660	4.0906	0.2793	NA	23.5877	7.8198	18.3657
1	4	11	1174.5	3.1368	4.0518	0.1862	NA	18.8897	5.4300	8.2523
1	4	11	1174.5	4.8946	6.3010	0.0580	5.7262	17.1514	0.1403	1.1639
1	4	11	1174.5	0.0309	0.0094	0.0170	3.3881	0.3884	0.0957	0.2721
2	4	11	1489.5	0.0328	0.0179	0.0266	6.6471	0.6735	0.0926	4.2793
2	4	11	1489.5	0.3401	9.6929	0.2301	7.9192	21.5154	6.4822	53.5149
2	4	11	1489.5	1.4695	7.9303	0.2744	9.2617	23.7989	5.9131	60.2298
3	4	11	1873.9	6.1314	6.5416	0.1103	10.5532	31.4425	5.1007	19.4203
3	4	11	1873.9	5.9240	7.1912	0.0560	3.5190	21.8970	0.3418	5.7273
3	4	11	1873.9	0.3981	2.8722	0.3820	4.9409	19.7233	10.8194	23.9874
1	4	12	1343	2.2031	2.8896	0.0459	5.1471	9.0582	0.1297	0.4196
1	4	12	1343	42.3709	9.7090	16.6096	0.0196	6.9920	1.0108	62.8664
1	4	12	1343	5.8560	8.8628	0.0549	10.5451	23.8877	0.1665	3.8829
2	4	12	1755	1.9917	7.2471	0.2841	7.7853	39.4541	10.3697	44.6040
2	4	12	1755	6.2126	7.6093	0.0606	3.5695	26.3286	1.0401	4.8970
2	4	12	1755	0.5250	5.2325	0.2448	6.7118	24.8085	8.7831	57.1686
1	10	1	47	0.0517	0.0106	0.0247	11.7691	0.3491	0.0440	0.2771
1	10	1	47	0.0479	0.0070	0.0232	10.9632	0.3305	0.0402	0.2706

1	10	1	47	NA	0.0076	0.0340	8.0499	0.3540	0.0437	0.2704
2	10	1	47.5	NA	0.0092	0.0386	7.6208	0.2650	0.0357	0.2601
2	10	1	47.5	0.0401	0.0124	0.0635	12.2938	0.3178	0.0370	0.2999
2	10	1	47.5	0.0146	0.0214	0.0845	10.7899	0.3704	0.0464	0.3853
4	10	1	48.3	0.0182	0.0096	0.0265	12.1101	0.2470	0.0487	0.2916
4	10	1	48.3	0.0203	0.0090	0.0271	9.9559	0.2544	0.0467	0.2771
4	10	1	48.3	NA	0.0088	0.0261	12.1414	0.2413	0.0517	0.3079
2	10	2	95.5	0.0505	0.0093	0.0417	11.4789	0.3097	0.0418	0.2892
2	10	2	95.5	0.0162	0.0121	0.0381	8.4182	0.3302	0.0393	0.2871
2	10	2	95.5	0.0119	0.0088	0.0418	7.9594	0.2956	0.0400	0.2695
3	10	2	96.5	0.1518	0.0095	0.0247	9.8704	0.3060	0.0433	0.2647
3	10	2	96.5	0.2324	0.0079	0.0212	7.6277	0.3035	0.0386	0.2774
3	10	2	96.5	0.0097	0.0081	0.0238	8.1114	0.2986	0.0417	0.2647
1	10	3	144.1	0.0509	0.0092	0.0173	10.7433	0.3509	0.0485	0.2825
1	10	3	144.1	0.0540	0.0086	0.0187	10.7938	0.3491	0.0461	0.2852
1	10	3	144.1	0.0173	0.0087	0.0269	7.8565	0.3914	0.0535	0.2937
2	10	3	143.7	0.0285	0.0115	0.0453	10.4603	0.3239	0.0429	0.3259
2	10	3	143.7	NA	0.0131	0.0621	6.7291	0.3429	0.0443	0.2893
2	10	3	143.7	0.0523	0.0103	0.0371	12.0538	0.3309	0.0406	0.3037
4	10	3	146	0.0160	0.0027	0.0144	7.1623	0.3004	0.0500	0.2122
4	10	3	146	0.0262	0.0023	0.0148	5.2960	0.3227	0.0500	0.2137
4	10	3	146	0.0262	0.0015	0.0156	11.0630	0.3002	0.0495	0.2199
2	10	4	191.5	0.0978	0.0111	0.0428	7.4584	0.3340	0.0476	0.2846
2	10	4	191.5	0.0147	0.0106	0.0367	7.4121	0.3614	0.0479	0.2847
2	10	4	191.5	0.0173	0.0108	0.0288	6.5067	0.2979	0.0416	0.2644
4	10	4	192.2	NA	0.0084	0.0182	7.5137	0.2422	0.0533	0.3001
4	10	4	192.2	NA	0.0026	0.0266	4.7357	0.5462	0.2175	NA
4	10	4	192.2	0.0485	0.0092	0.0181	10.6959	0.2502	0.0553	0.2867
1	10	5	239	1.5933	1.6212	0.5846	4.7872	7.4741	0.0566	1.6593
1	10	5	239	0.0523	0.0090	0.0176	11.0633	0.5051	0.0563	0.2833
1	10	5	239	NA	0.0171	0.0529	8.8222	0.8917	0.2131	0.9450
2	10	5	239.5	0.0575	0.0115	0.0447	6.2327	0.7648	0.0453	0.2797
2	10	5	239.5	0.0177	0.0181	0.0506	7.8895	0.3967	0.0551	0.3250
2	10	5	239.5	0.0157	0.0137	0.0349	6.4648	0.5909	0.0484	0.2708
2	10	6	264.5	0.0375	0.0393	0.0334	6.5510	0.7461	0.0516	0.2847
2	10	6	264.5	NA	0.0110	0.0312	6.5747	0.5766	0.0649	0.2827
2	10	6	264.5	0.2993	0.4860	0.0283	10.4715	2.6964	0.0516	0.4882
3	10	6	264.5	0.5487	0.5060	0.0331	4.4801	3.8112	0.0608	2.4363
3	10	6	264.5	0.2177	0.0098	0.0166	6.8192	0.3096	0.0530	0.2681
3	10	6	264.5	0.2397	0.3545	0.0283	7.0860	3.1460	0.0563	2.0405
4	10	6	275.5	NA	0.2321	0.0836	7.4099	1.4631	0.2218	NA
4	10	6	275.5	0.0988	0.1719	0.0200	9.4073	1.6655	0.0614	0.3528
4	10	6	275.5	NA	0.0131	0.0464	7.1131	0.7021	0.4027	NA
1	10	7	288	1.1936	1.4049	0.0340	10.2120	5.3418	0.0531	0.4887
1	10	7	288	0.0120	0.0109	0.0277	6.9596	0.3584	0.0628	0.2798
1	10	7	288	4.4022	3.4816	1.7770	3.4099	8.3313	0.0713	1.9204
2	10	7	288.5	0.6128	0.8989	0.0354	5.9804	4.0807	0.0544	0.4473
2	10	7	288.5	0.7585	0.9015	0.3389	11.6003	5.4812	0.0622	308.2861
2	10	7	288.5	0.6974	0.8790	0.0473	5.9631	4.1938	0.0539	0.4169
3	10	7	288.1	0.5570	0.5426	0.0368	6.8016	4.1688	0.0662	2.5556
3	10	7	288.1	0.1993	0.0098	0.0152	6.2962	0.2812	0.0566	0.2723
3	10	7	288.1	0.0157	0.0143	0.0233	8.9721	0.3653	0.0565	0.3511
4	10	7	288.6	0.4484	0.7165	0.0232	7.3472	3.2642	0.0630	0.4204
4	10	7	288.6	0.1353	0.1306	0.0223	3.9552	1.9913	0.2446	0.4799
4	10	7	288.6	NA	0.0042	0.0347	6.4519	1.7907	0.2789	NA
1	10	8	311.2	0.0244	0.0115	0.0202	4.9261	0.1691	0.0546	0.2976
1	10	8	311.2	1.1944	1.2590	0.0324	5.1460	5.0474	0.0513	0.5862
1	10	8	311.2	1.4659	1.6253	0.0451	5.1035	5.8989	0.0698	0.4865
2	10	8	311.5	1.3405	1.5315	0.0625	5.8681	6.0256	0.0614	0.3573
2	10	8	311.5	1.2634	1.2740	0.0451	7.7765	6.9729	0.0587	2.5943
2	10	8	311.5	1.9274	1.9472	10.0131	0.1072	80.6290	0.0542	10.4399
3	10	8	313.5	0.2190	0.0101	0.0162	5.0605	0.3289	0.0561	0.2641
3	10	8	313.5	0.5236	0.6737	0.0248	7.5908	2.4176	0.0507	0.3209

3	10	8	313.5	0.0189	0.0132	0.0373	6.6635	0.3528	0.0676	0.2827
4	10	8	313.2	1.4577	1.8326	0.0289	5.5953	6.5812	0.0593	0.5991
4	10	8	313.2	1.3251	1.7070	0.0781	NA	6.9124	0.9653	NA
4	10	8	313.2	0.5713	0.5522	0.1037	5.7770	2.1333	0.0739	0.9199
1	10	9	336.6	1.6650	1.7144	0.0437	10.1745	6.4945	0.0621	0.6784
1	10	9	336.6	2.5286	2.2740	0.0761	5.6609	7.5720	0.0627	0.4356
2	10	9	336.7	0.8964	0.8436	0.2819	10.1644	4.1315	0.9337	262.3336
2	10	9	336.7	1.5947	1.4721	0.0426	4.1970	5.6157	0.0537	0.4804
2	10	9	336.7	0.7587	0.8825	0.3108	11.8852	5.6365	0.0578	301.4754
3	10	9	335.6	2.3050	2.0725	0.0440	4.6128	7.0203	0.0632	0.5207
3	10	9	335.6	0.9627	1.2031	0.0247	8.1894	3.8720	0.1291	0.1986
3	10	9	335.6	0.6532	0.7214	0.0235	5.8638	3.7434	0.0672	0.5713
4	10	9	337.1	1.4598	1.7131	0.0294	5.8255	6.2523	0.0719	0.6205
4	10	9	337.1	1.5673	1.5365	0.0318	10.8274	5.6084	0.0541	0.4190
4	10	9	337.1	2.1146	1.8787	0.1117	6.0499	6.9552	0.7998	NA
1	10	10	359	26.3722	9.4241	2.8609	NA	11.7403	0.2380	5.1169
1	10	10	359	2.8081	2.5484	0.0774	6.1033	7.7695	0.0689	0.4916
1	10	10	359	2.4894	2.2228	0.0555	4.2890	7.7040	0.0644	0.5116
3	10	10	359.7	2.0605	2.0493	0.0473	10.6569	7.0151	0.0654	0.8741
3	10	10	359.7	2.1564	1.7988	0.0407	3.4376	6.6024	0.0516	0.6327
3	10	10	359.7	0.0129	0.0088	0.0277	8.3796	0.1195	0.0750	0.2884
4	10	10	360.5	2.2845	2.1068	0.0425	5.6600	6.5367	0.0729	0.6676
4	10	10	360.5	2.7781	3.0264	0.0911	2.8358	13.7964	1.3235	1.5116
4	10	10	360.5	2.2939	2.1025	0.1052	5.2189	8.0450	0.7969	NA
1	10	11	382.9	3.4250	2.9287	0.0560	2.3127	9.4580	0.0730	0.8552
1	10	11	382.9	11.4386	5.0381	4.6850	1.4485	0.7837	0.2086	2.5120
1	10	11	382.9	2.2257	1.8824	0.0572	2.1603	6.1983	0.0634	0.4238
3	10	11	408	5.0337	3.9449	0.1171	1.4314	10.8233	0.0718	0.5698
3	10	11	408	3.8608	2.3935	0.0813	4.5348	5.0079	0.1374	0.2168
3	10	11	408	NA	0.0120	0.0526	6.7469	0.1966	0.0674	0.2672
2	15	1	23.6	0.0094	0.0099	0.0534	12.6178	0.3019	0.0379	0.3754
2	15	1	23.6	0.0121	0.0112	0.0874	10.5465	0.3219	0.0352	0.3196
2	15	1	23.6	0.0369	0.0124	0.0615	10.3415	0.3077	0.0379	0.2934
3	15	1	23.5	0.2083	0.0101	0.0337	12.1519	0.3288	0.0395	0.2912
3	15	1	23.5	0.0090	0.0081	0.0312	8.8048	0.3028	0.0374	0.2792
3	15	1	23.5	0.1232	0.0071	0.0220	10.3379	0.2872	0.0394	0.2755
4	15	1	24.1	0.0190	0.0103	0.0315	10.2161	0.2760	0.0472	0.2746
4	15	1	24.1	NA	0.0091	0.0274	10.0738	0.2495	0.0471	0.2824
4	15	1	24.1	0.0180	0.0086	0.0321	11.1905	0.2664	0.0464	0.2860
1	15	2	47	0.0133	0.0087	0.0331	7.3660	0.3701	0.0465	0.2923
1	15	2	47	0.0719	0.0076	0.0234	11.7237	0.3423	0.0448	0.2758
1	15	2	47	0.0405	0.0079	0.0238	11.7566	0.3449	0.0422	0.2769
2	15	3	71.5	0.0146	0.0100	0.0387	7.5081	0.4995	0.0414	0.2914
2	15	3	71.5	0.0098	0.0100	0.0409	12.3695	0.4289	0.0414	0.3037
2	15	3	71.5	0.0501	0.0102	0.0490	7.0156	0.3404	0.0408	0.2867
3	15	3	71.5	0.1241	0.0082	0.0210	9.6397	0.3679	0.0370	0.2451
3	15	3	71.5	0.0197	0.0086	0.0245	8.5119	0.6080	0.0421	0.2781
3	15	3	71.5	0.2505	0.0082	0.0204	7.7391	0.4942	0.0449	0.2718
4	15	3	73	0.0251	0.0040	0.0177	11.8524	0.3951	0.0406	0.3554
4	15	3	73	NA	NA	0.0157	7.7695	0.2645	0.0429	0.2229
4	15	3	73	0.0203	0.0035	0.0185	7.8608	0.3350	0.0450	0.2710
1	15	4	79	0.0109	0.0084	0.0255	7.4195	0.4047	0.0478	0.2680
1	15	4	79	0.0265	0.0102	0.0254	12.0001	0.4257	0.0521	0.3048
1	15	4	79	0.0387	0.0095	0.0215	12.0251	0.5018	0.0483	0.2898
2	15	4	79.4	NA	0.0113	0.0413	12.0945	0.3241	0.0411	0.3159
2	15	4	79.4	0.0103	0.0136	0.0627	9.9836	0.7232	0.0425	0.2885
2	15	4	79.4	0.0955	0.0655	0.0849	8.8200	2.2791	0.0436	11.5702
3	15	4	79.6	0.0483	0.0795	0.0338	7.9925	1.8123	0.0432	1.1329
3	15	4	79.6	0.2303	0.0077	0.0184	6.0704	0.5167	0.1018	0.4066
3	15	4	79.6	NA	0.0122	0.0460	6.9059	0.5279	0.0450	0.2741
4	15	4	79.7	0.0291	0.0186	0.0227	11.8764	0.8565	0.0541	0.4947
4	15	4	79.7	0.0722	0.1026	0.0268	7.3186	1.7485	0.0533	1.4132
4	15	4	79.7	0.0508	0.0600	0.0257	5.8035	1.4343	0.0564	0.9809

2	15	5	91.7	0.0452	0.0559	0.0738	6.0576	1.2077	0.0456	1.1648
2	15	5	91.7	0.0835	0.0641	0.0505	12.0422	1.7509	0.0443	1.3626
2	15	5	91.7	0.0361	0.0132	0.0497	6.7193	0.7786	0.0421	1.2497
3	15	5	91.5	0.2637	0.1065	0.0389	10.5241	2.2072	0.0472	1.5963
3	15	5	91.5	0.0215	0.0234	0.0268	7.3611	1.4340	0.0479	0.5816
3	15	5	91.5	0.0309	0.0363	0.0572	6.6864	1.4173	0.0449	0.7709
4	15	5	92	0.0315	0.0145	0.0226	11.7856	0.2767	0.0530	0.4671
4	15	5	92	0.3543	0.3619	0.0355	6.7846	2.4640	0.1730	3.0842
4	15	5	92	0.3735	0.2865	0.0410	6.7510	2.6050	0.1214	3.2366
1	15	6	103	0.0462	0.0537	0.0329	6.7458	0.7977	0.0518	0.9054
1	15	6	103	0.0306	0.0249	0.0255	11.7346	0.8388	0.0509	0.5224
1	15	6	103	0.2573	0.2967	0.0403	5.7820	3.2067	0.0480	2.3481
2	15	6	103.4	0.0702	0.1015	0.0448	5.7412	1.1559	0.0449	1.5793
2	15	6	103.4	0.3934	0.3919	0.0554	5.6979	2.7805	0.0490	2.7170
2	15	6	103.4	0.0222	0.0401	0.0651	9.6225	0.3188	0.0429	0.4196
3	15	6	104	0.3563	0.1889	0.0501	10.2879	2.9718	0.0480	4.7069
3	15	6	104	0.2197	0.3083	0.0407	7.1721	2.5644	0.0521	3.3836
3	15	6	104	0.5951	0.6127	0.0725	6.2299	4.6218	0.0556	4.6334
4	15	6	104	0.3490	0.3918	0.0499	7.1656	3.9345	0.0627	3.8506
4	15	6	104	0.2471	0.3391	0.0422	4.4464	3.1862	0.0629	2.9287
4	15	6	104	0.0988	0.1018	0.0266	5.6654	1.1244	0.1553	0.6757
1	15	7	116	0.1492	0.3020	0.0358	6.0763	1.7076	0.0450	1.6170
1	15	7	116	0.1403	0.1675	0.0398	11.8778	1.4774	0.0453	2.3149
1	15	7	116	0.1433	0.2465	0.0314	5.7179	1.6007	0.0544	1.5493
2	15	7	115.8	0.4307	0.4984	0.0711	6.8450	3.5048	0.0498	4.6621
2	15	7	115.8	0.1368	0.2143	0.0853	6.8449	1.5849	0.0493	2.0880
2	15	7	115.8	0.6498	0.8545	0.0637	5.5460	4.6039	0.0537	4.7401
3	15	7	115.6	0.6645	0.7277	0.0583	6.1059	4.7523	0.0617	4.2107
3	15	7	115.6	0.6621	0.7310	0.0746	5.1943	4.9195	0.0575	4.1714
4	15	7	116.3	0.3818	0.5651	0.0400	6.8810	3.3810	0.0586	3.7203
4	15	7	116.3	0.4986	0.6972	0.0468	5.8076	5.1327	0.0626	4.9118
1	15	8	127.3	0.5186	0.6283	0.0862	7.1539	4.4227	0.0805	5.5785
1	15	8	127.3	0.4128	0.4996	0.0546	10.8430	3.3678	0.0587	5.5843
1	15	8	127.3	0.2949	0.3863	0.0525	6.3863	2.8801	0.0561	4.1646
2	15	8	127.2	0.8042	0.9197	0.0641	3.8500	4.3793	0.0624	4.0857
2	15	8	127.2	0.9035	0.7744	0.2370	5.9133	4.8046	0.0555	128.2494
2	15	8	127.2	0.4828	0.5637	0.0599	5.9730	3.3995	0.0485	3.7256
4	15	8	129.5	0.5485	0.5819	0.0865	NA	5.0844	0.1147	4.9748
4	15	8	129.5	1.1493	1.2313	0.0557	6.6632	5.4729	0.0640	4.6068
4	15	8	129.5	0.5037	0.5164	0.0744	6.5319	3.0277	0.0630	5.1693
1	15	9	140	0.8399	0.7318	0.3105	11.3309	4.3715	0.0766	197.6594
1	15	9	140	0.4565	0.4977	0.0572	10.3751	3.2252	0.0649	5.1455
1	15	9	140	0.7745	0.8583	0.0772	6.6272	5.4243	0.0830	4.3121
1	15	10	144.1	1.2559	1.1543	0.0967	1.7226	5.5294	0.0846	3.9570
1	15	10	144.1	0.8752	1.0062	0.0606	10.8785	5.1307	0.0679	3.0777
1	15	10	144.1	0.4192	0.5789	0.0504	6.0535	2.9246	0.0608	3.4077
2	20	1	11.4	0.0127	0.0127	0.0888	11.5394	0.3223	0.0332	0.2851
2	20	1	11.4	0.0119	0.0085	0.0563	12.6008	0.3006	0.0380	0.2929
2	20	1	11.4	0.0295	0.0089	0.0541	14.5710	0.2910	0.0378	0.3128
4	20	1	11.9	NA	0.0084	0.0282	12.7768	0.2438	0.0455	0.2735
4	20	1	11.9	0.0312	0.0096	0.0385	13.1906	0.3136	0.0472	0.3157
4	20	1	11.9	0.0215	0.0086	0.0343	10.9213	0.2534	0.0448	0.2823
1	20	2	17.3	0.0419	0.0082	0.0251	12.3930	0.3573	0.0426	0.2879
1	20	2	17.3	NA	0.0073	0.0381	7.7858	0.3800	0.0437	0.3160
1	20	2	17.3	0.0385	0.0071	0.0272	12.0658	0.3480	0.0404	0.2797
2	20	2	18.5	NA	0.0086	0.0429	7.1781	0.2942	0.0357	0.3125
2	20	2	18.5	NA	0.0100	0.0531	12.4824	0.3036	0.0368	0.2931
2	20	2	18.5	NA	0.0081	0.0540	12.5519	0.3015	0.0366	0.2898
3	20	2	18.5	0.1862	0.0085	0.0352	12.2438	0.3210	0.0402	0.2851
3	20	2	18.5	0.1775	0.0078	0.0246	8.1290	0.2938	0.0368	0.2639
3	20	2	18.5	NA	0.0083	0.0327	11.4451	0.3077	0.0400	0.2872
2	20	3	23.6	0.0335	0.0099	0.0455	10.8921	0.3022	0.0385	0.3036
2	20	3	23.6	0.0088	0.0149	0.0843	11.7495	0.3471	0.0389	0.2923

2	20	3	23.6	NA	0.0097	0.0519	12.3935	0.3073	0.0353	0.2949
4	20	3	24.1	0.0154	0.0082	0.0260	8.7713	0.2430	0.0448	0.2645
4	20	3	24.1	0.0261	0.0081	0.0265	12.0164	0.2419	0.0471	0.2820
4	20	3	24.1	0.0180	0.0072	0.0300	9.8039	0.2538	0.0483	0.2768
1	20	4	29	0.0439	0.0085	0.0242	11.6462	0.3408	0.0418	0.2784
1	20	4	29	NA	0.0093	0.0299	8.1284	0.3929	0.0468	0.2844
1	20	4	29	0.0371	0.0076	0.0240	11.3899	0.3450	0.0409	0.2799
2	20	4	29.5	NA	0.0105	0.0548	13.2698	0.3364	0.0401	0.3138
2	20	4	29.5	0.0265	0.0093	0.0432	10.6068	0.3014	0.0400	0.2920
2	20	4	29.5	0.0142	0.0098	0.0483	11.0323	0.3376	0.0367	0.3128
3	20	4	29.5	0.2036	0.0085	0.0320	12.1603	0.3448	0.0422	0.2873
3	20	4	29.5	0.1585	0.0073	0.0244	9.2846	0.3118	0.0402	0.2709
3	20	4	29.5	0.0109	0.0079	0.0307	9.3907	0.3342	0.0421	0.2705
1	20	5	36.3	0.0211	0.0222	0.0367	5.8015	1.3715	0.0440	0.5796
1	20	5	36.3	0.0158	0.0073	0.0293	7.3027	0.5971	0.0450	0.2802
1	20	5	36.3	0.0184	0.0106	0.0308	12.5649	0.6057	0.0478	0.3070
2	20	5	35.3	0.0305	0.0093	0.0477	11.5934	0.6849	0.0424	1.4095
2	20	5	35.3	0.0126	0.0151	0.0744	11.6131	0.3811	0.0405	0.2944
2	20	5	35.3	0.0146	0.0128	0.0482	7.1654	0.3673	0.0413	0.3074
3	20	5	35.4	0.1717	0.0157	0.0325	12.2347	0.8548	0.0442	0.3612
3	20	5	35.4	0.1281	0.0082	0.0233	6.2971	0.8449	0.0432	0.2627
3	20	5	35.4	0.0106	0.0091	0.0294	8.8393	0.4341	0.0414	0.2719
4	20	5	35.9	NA	0.0111	0.0243	8.0735	0.7817	0.0486	0.2791
4	20	5	35.9	0.0249	0.0087	0.0264	7.5552	0.6259	0.0507	0.2879
4	20	5	35.9	0.0162	0.0089	0.0251	8.5682	0.2940	0.0492	0.2933
1	20	6	47	0.0604	0.0846	0.0482	7.8642	1.1580	0.0515	1.4114
1	20	6	47	0.2502	0.3057	0.0552	5.7974	3.7935	0.0519	4.9681
1	20	6	47	0.0832	0.1098	0.0383	11.9592	1.8794	0.0482	1.9580
2	20	6	42.5	0.0492	0.0707	0.0803	10.9889	1.8437	0.0397	1.4752
2	20	6	42.5	0.0177	0.0167	0.0478	7.6089	0.6357	0.0385	0.3829
2	20	6	42.5	0.0175	0.0244	0.0648	12.7651	1.4962	0.0413	6.9100
4	20	6	43	0.0784	0.0866	0.0299	11.3839	1.9064	0.0485	1.2769
4	20	6	43	0.0446	0.0351	0.0312	7.3373	1.4305	0.0522	0.8865
4	20	6	43	0.0592	0.0683	0.0281	5.8990	1.3221	0.0524	1.0046
1	20	7	53	0.3511	0.3853	0.0665	6.1392	3.2851	0.0505	4.9935
1	20	7	53	0.2067	0.2529	0.0552	12.5121	2.3895	0.0531	4.6085
2	20	7	47.5	0.2372	0.2483	0.0587	8.3162	3.5244	0.0456	4.3269
2	20	7	47.5	0.1028	0.1154	0.0621	7.6389	1.7987	0.0436	2.2235
2	20	7	47.5	0.0907	0.1144	0.0819	10.2842	1.5477	0.0390	1.9541
3	20	7	47.4	0.2855	0.1350	0.0373	10.2084	1.9266	0.0448	2.0175
3	20	7	47.4	0.2684	0.1246	0.0380	6.5516	3.0910	0.0410	2.6644
3	20	7	47.4	0.1513	0.1610	0.0494	5.7102	2.5465	0.0407	2.3963
4	20	7	48.3	0.6597	0.8294	0.0815	6.7328	5.5371	0.0669	3.6739
4	20	7	48.3	0.2152	0.2559	0.0345	11.8902	2.6083	0.0419	2.9855
4	20	7	48.3	0.1174	0.1590	0.0320	5.5954	2.4950	0.0457	2.2409
1	20	8	59	0.4480	0.5404	0.0688	5.7273	3.5361	0.0542	6.0698
1	20	8	59	0.5188	0.5863	0.0987	7.8590	4.9634	0.0667	4.8165
1	20	8	59	12.2790	7.4238	1.4679	9.8854	37.9358	0.0642	35.3954
2	20	8	77.5	0.2789	0.2543	0.0773	2.0928	3.0972	0.0427	3.8198
2	20	8	77.5	0.4902	0.4394	0.0922	6.4054	3.4157	0.0534	4.5210
2	20	8	77.5	0.7866	0.9851	0.4302	12.1142	5.5637	0.0566	319.0682
3	20	8	53.5	0.5036	0.3997	0.0596	11.7776	3.6616	0.0474	5.3201
3	20	8	53.5	0.2549	0.2856	0.0508	7.5768	3.4193	0.0504	3.9775
3	20	8	53.5	0.1751	0.2028	0.0708	7.4095	2.6954	0.0471	4.0361
4	20	8	54	0.1090	0.1639	0.0354	8.6488	1.3535	0.0519	1.5819
4	20	8	54	0.5890	0.6983	0.0611	4.5769	5.0459	0.0562	5.2401
4	20	8	54	0.5504	0.5387	0.0655	5.7853	4.2233	0.0504	5.2311
1	20	9	66	0.6342	0.8056	0.0859	6.9291	4.8687	0.0696	5.6262
1	20	9	66	4.8121	6.3486	1.8982	10.2057	22.4674	0.0556	7.6803
1	20	9	66	0.7738	0.8228	0.0921	NA	5.1963	0.0758	4.6374
3	20	9	59.4	0.6888	0.5725	0.0706	6.1571	4.4887	0.0548	5.4438
3	20	9	59.4	0.6268	0.7915	0.0837	7.0302	5.0949	0.0636	5.0043
3	20	9	59.4	0.3353	0.4106	0.0864	8.5735	3.3133	0.0503	6.2832

4	20	9	60.3	0.5084	0.5232	0.0622	6.5614	4.0555	0.0590	5.7420
4	20	9	60.3	0.6781	0.7194	0.0771	1.5689	5.0935	0.0576	4.6931
4	20	9	60.3	0.4085	0.5913	0.0552	6.3420	3.9224	0.0605	4.6255
1	20	10	71.2	0.8892	0.9500	0.0733	5.8336	4.6132	0.0596	5.9420
1	20	10	71.2	0.6626	0.6897	0.0935	2.6948	4.5263	0.0860	5.2718
1	20	10	71.2	0.5641	0.6261	0.0832	2.1030	3.9678	0.0709	5.1398
3	20	10	66.5	0.9638	0.6661	0.0915	2.7819	3.8258	0.0698	5.4741
3	20	10	66.5	0.6063	0.5134	0.0971	5.7452	4.0224	0.0538	5.0846
3	20	10	66.5	0.6202	0.7386	0.1035	7.2449	5.2542	0.0937	5.3980
